# From Skin Barrier Dysfunction to Systemic Impact of Atopic Dermatitis: Implications for a Precision Approach in Dermocosmetics and Medicine

**DOI:** 10.3390/jpm12060893

**Published:** 2022-05-28

**Authors:** Laura Maintz, Thomas Bieber, Helen D. Simpson, Anne-Laure Demessant-Flavigny

**Affiliations:** 1 Department of Dermatology and Allergy, University Hospital Bonn, 53127 Bonn, Germany; thomas.bieber@ukbonn.de; 2 Christine Kühne Center for Allergy Research and Education Davos (CK-CARE), 7265 Davos, Switzerland; 3 Davos Biosciences, Herman-Burchard-Str. 9, CH-7265 Davos Wolfgang, Switzerland; 4 My Word Medical Writing, 13260 Cassis, France; helen@mywordmedicalwriting.com; 5 La Roche-Posay International, 92300 Levallois-Perret, France; anne-laure.demessant@loreal.com

**Keywords:** asthma, atopic dermatitis, atopic march, biologic therapies, comorbidities, dermocosmetics, emollient, immunity, precision medicine, systemic disease

## Abstract

Atopic dermatitis (AD) affects up to 20% of children and is considered the starting point of the atopic march with the development of food allergy, asthma, and allergic rhinitis. The heterogeneous phenotype reflects distinct and/or overlapping pathogenetic mechanisms with varying degrees of epidermal barrier disruption, activation of different T cell subsets and dysbiosis of the skin microbiome. Here, we review current evidence suggesting a systemic impact of the cutaneous inflammation in AD together with a higher risk of asthma and other comorbidities, especially in severe and persistent AD. Thus, early therapy of AD to restore the impaired skin barrier, modified microbiome, and target type 2 inflammation, depending on the (endo)phenotype, in a tailored approach is crucial. We discuss what we can learn from the comorbidities and the implications for preventive and therapeutic interventions from precision dermocosmetics to precision medicine. The stratification of AD patients into biomarker-based endotypes for a precision medicine approach offers opportunities for better long-term control of AD with the potential to reduce the systemic impact of a chronic skin inflammation and even prevent or modify the course, not only of AD, but possibly also the comorbidities, depending on the patient’s age and disease stage.

## 1. Introduction

Atopic dermatitis (AD) is the most common chronic inflammatory skin disorder with a prevalence of 15–20% in children and up to 10% in adults [1,2]. Thus, AD affects not on-ly the individual patient’s health and quality of life, but also represents a significant bur-den on the health system [3]. The inherent complexity of genetic factors, environmental influences, skin barrier dysfunction, immune dysregulation, microbial dysbiosis, as well as the resulting cutaneous inflammation and its potential systemic impact, are not fully understood. Furthermore, the complex pathophysiology of AD may lead to atopic and non-atopic comorbidities. AD is considered the starting point of the atopic march, leading to the development of comorbidities such as asthma, food allergies (FA), and allergic rhinitis (AR) [4,5].

Experiments in different mouse models [6,7] have suggested that genetics, epigenetics, and non-type 2 unspecific inflammation from the innate immune response likely con-tribute to the pathophysiologic makeup of the initial phase of AD in the early years. Loss of barrier integrity has been hypothesized to enable penetration of allergens, pollutants and microbes, and initiation of an inflammatory immune cascade of events leading to sensitization and a proinflammatory atopic state. Both epidermal barrier dysfunction and type 2 (T2) inflammation are tightly associated with AD lesions and provide an important background for sensitization. Evidence suggests that both epidermal barrier dysfunction and activation of the local innate immune system (due to gene mutations, allergens, pol-lution, scratching, microbiome alterations) induce a pro-T2 microenvironment locally and in the draining lymph nodes and a core T2 immune response (the so-called T2 inflammation). This sequence plays a central role in AD and is suspected to be instrumental for the atopic march that may start from as early as 3 months of age [8]. A mixed response or kind of immunological march, involving Th22, Th1, Th17, immune cells, and other types of re-sponses may occur, especially in the chronic phase [9,10,11].

AD has generally been considered a single disease that is treated with a one-size-fits-all standard of care. However, current evidence is beginning to elucidate the heterogeneity of the phenotypes (such as age at onset, triggers, comorbidities, physiological traits, ethnic background, inflammation types, and treatment responses) with mechanistic, clinical and translational consequences. Different phenotypes may reflect distinct and/or overlapping pathogenetic mechanisms with varying degrees of epidermal barrier disruption, activation of different T cell subsets, and dysbiosis of the skin microbiota. Moving from clinical to molecular approaches to define endotypes could now lead to more targeted and personalized approaches to AD therapy.

Epidermal barrier dysfunction has two main origins: an intrinsic genetic background and an underlying inflammation. The filaggrin gene mutation has a key role in epidermal integrity and AD, while an increase in epithelial barrier-damaging agents, e.g., pollution, diet, and stress, may explain the rise in AD incidence as nations become more industrialized [12,13]. Conversely, the amelioration of AD symptoms and barrier function with biologics that target individual inflammatory pathways by targeting T2 immune responses support the role of inflammation in the alteration of the barrier function. The allergen exposure hypothesis assumes that allergens penetrate through the impaired skin barrier, where in the presence of cytokine dysregulation, they promote T2 inflammation, which might lead to clinical FA, whilst early life oral exposure is protective [14,15,16]. Immune dysfunction and itch may further exacerbate the impaired skin barrier to form a vicious cycle [17] and reinforce the immune response.

In this manuscript we review current evidence suggesting AD has a systemic impact, due to the substantial inflammatory burden in the skin with consequences on the immune response and potentially other organs. We also discuss what we can learn from the comorbidities with a focus on asthma and the implications for preventive and therapeutic interventions from precision dermocosmetics to precision medicine.

## 2. Evidence That Skin Barrier Dysfunction Is a Key Precursor of AD

The skin barrier functions (physical, immune, chemical, microbiome barriers) of the epidermis are crucial for the protection against pathogens, allergens, toxins, and other irritants and maintenance of pH, hydration, and antimicrobial functions. In AD, the epidermal barrier dysfunction has two origins.

Firstly, a major predisposing factor for early-onset, severe, and long-lasting AD is that mutations in genes encoding epidermal structural proteins (e.g., Filaggrin-1 or 2, Claudin-1) cause functional impairments of tight junctions and barrier dysfunction. These are thought to play an essential role in the initiation of early-onset AD [18]. Additionally, mutations in proteases and protease inhibitors, serine peptidase inhibitor Kazal-type 5 (SPINK5), and corneodesmosin, lead to altered desquamation and defects in the skin barrier [6,19]. In AD, the magnitude of skin barrier dysfunction, which manifests as dry skin and increased transepidermal water loss (TEWL), correlates with AD disease severity [20]. Multiple factors, including immune dysregulation, genetic mutations encoding tight junction structures, deficiency of antimicrobial peptides, and skin dysbiosis, contribute to skin barrier defects [21]. There is an increasing body of evidence showing that disruption of the skin barrier function can promote an antigen-independent inflammation and T cell infiltration as a downstream consequence of a sustained, barrier-driven cytokine cascade [22,23]. Skin barrier disruption can trigger inflammation as damaged keratinocytes release thymic stromal lymphopoietin (TSLP), as well as the alarmins IL-33, IL-25, uric acid, ATP, HMGB1, and S100 proteins [24]. These factors create a pro-T2 inflammatory environment in the skin and favor the development of IgE-mediated sensitization in the regional lymph nodes [6].

Secondly, the local inflammatory reaction, either from the initial innate immune response or as a result of the adaptive immune response, reciprocally contributes to the skin barrier defect in AD [25]. The immune response in AD is predominantly characterized by core T helper (Th) 2 cell-mediated pathways. In addition to localized skin lesions, patients with AD can exhibit signs of systemic immune dysregulation, as demonstrated by peripheral eosinophilia, unbalanced levels of Th cells, and increased serum Immunoglobulin (Ig) E levels [26]. Mild and limited AD show high levels of Th2/Th22 cell activation primarily in skin lesions and lacks the systemic inflammation of moderate and severe disease [27]. Fifty percent of AD patients exhibit a T2 high endotype that is characterized by more severe disease [28]. Systemic activation of other multiple Th cell subsets in AD, in addition to simply Th1–Th2, has been observed [29]. In AD, inflammation is linked to elevated levels of inflammatory cytokines, including T2-associated IL-4, -13 [30,31], -31, but also IL-22, Th17, and Th1-associated interferon-gamma, with downstream activation of the Janus kinase (JAK)-signal transducer and activator of transcription (STAT) pathway [32]. Most importantly, there is a clear correlation between the systemically measurable cytokines, such as TARC/CCL-17, IL-13 or IL-22, and the clinical severity of AD [33]. Since also the non-lesional skin in moderate and severe AD has been shown to include substantial inflammation [34,35,36], it is assumed that even in less severe forms of AD, the overall systemic inflammatory burden originates in the skin.

### 2.1. Role of Microbiota in the Inflammation Driven by the Barrier Dysfunction

Immune responses to dysbiotic microbiota (in particular, overgrowth of *Staphylococcus aureus* *(S.a.)* together with reduced local bacterial diversity [5]) that cross the damaged skin barrier may be involved in the development of AD [37]. Up to 90% of patients with AD have *S.a*. colonization which correlates with AD severity, contributes to various pathophysiological factors such as skin barrier dysfunction through protease activity, downregulation of terminal differentiation markers in the skin, and production of virulence factors such as cytolysins, protein A, and *S.a.* superantigens, with upregulation of Th2 cytokines [5,16,38]. These Th2 cytokines inhibit the production of antimicrobial peptides in a vicious circle [16,39]. Staphylococcal-derived superantigens, such as *staphylococcal enterotoxin B* (*SEB*), could be shown to perpetuate cutaneous type 2 inflammation in response to peanut allergens and contribute to the development of a peanut allergy (thus contributing to a food allergy) [5,25,40]. Host-microbe interactions depend on the state of immune activation, host genetic predisposition, barrier status, microbe localization, and microbe–microbe interactions [41]. Microbiome dysbiosis causing inappropriate immune responses may be responsible for the initial stages of the atopic march that drives inflammation [42]. The interaction between the host (epidermis) and microbes is bidirectional, and it remains unclear whether microbiome dysbiosis, especially *S.a*. colonization, is the result or the reason for barrier impairment and inflammation [43]. Uncertainty also exists around whether the skin microbiota play a crucial role in the immune system and the inflammatory reaction in the later stages of AD beyond childhood. However, it is clear that epidermal Langerhans cells, which bridge the innate and adaptive immune systems in the recognition of potential pathogenic bacteria, are tolerized to *S.a*.-derived signals [43]. This phenomenon may contribute, at least in part, to the uncontrolled overgrowth of these bacteria and to the well-known dysbiosis.

### 2.2. Neuroinflammation and Itch

Pruritus is a key symptom of AD frequently contributing to impaired quality of life and sleep loss [44]. Pruritogens can bind to receptors on cutaneous primary sensory nerves (slow-conducting C and fast Aδ fibers) which mediate itch and pain [2,36]. Thus, patients frequently suffer not only from itch, but also from pain, burning, and stinging in lesional skin [45]. Scratching can perpetuate skin barrier damage and itch [32], leading to a vicious itch–scratch cycle. Crosstalk between keratinocytes, the immune system, and non-histaminergic sensory nerves is assumed to be key in the generation of chronic itch in AD [46]. Type 2 cytokines directly activate sensory neurons in both mice and humans, while chronic itch is dependent on neuronal IL-31R, IL-4Rα, and JAK1 signaling [47]. Neuropeptides (e.g., substance P or calcitonin gene-related peptide) released from immune (or non-immune skin barrier and microbiome) skin cells may be indirectly involved in the propagation of inflammation and itch [46,48]. Although histamine is a main mediator of allergic inflammation and itch, less than 5% of skin C-nerve fibers are histamine-sensitive [2], and therapies aimed at blocking histamine 1 receptors (H1R) have been largely ineffective for AD [2]. However, antagonists targeting the H4R exerting immunoregulatory effects on leukocytes are in clinical trials [3]. The H4R antagonist adriforant slightly improved disease severity without statistically significant effects on pruritus in a phase IIa study [49], and further programs for H4R antagonists for AD have been suspended [3,49]. The anti-IL-31 ab nemolizumab significantly reduced pruritus in AD and prurigo nodularis [50,51]. IL-31 is produced by several T2 cells such as Th2 cells, mast cells, macrophages, and dendritic cells, activates neurons via IL-31Rα, TRPV1 or TRPA1, and further induces the recruitment of T cells by chemotactic cytokines along with increases of neuronal branching in the skin in a vicious cycle [3,36,52].

The neuroimmune axis provides new insight into the molecular mechanisms of epithelial cells, immune cells, and nervous system communication. There is evidence that pruritogens are not only responsible for eliciting pruritus, but also interact with immune cells and act as inflammatory mediators, which exacerbate the severity of AD [53].

## 3. Comorbidities of AD

AD, especially the severe forms with early onset [54], has been associated with an increasing number of comorbidities, especially asthma and FA [8]. The progression from AD in early infancy to FA, asthma, and AR, i.e., atopic comorbidities, has been termed “atopic march” [5]. Serum biomarker profiles show that the comorbidities potentially share some common pathological mechanisms, suggesting that AD is a disease with systemic impact [9,55].

AD typically starts in infancy or early childhood with spontaneous disease clearance before adolescence in about 60% of the cases. However, there is growing evidence for AD as a lifelong disease with variable phenotypic expression, and a high rate of adult-onset or relapsing AD after long asymptomatic intervals [56,57,58,59,60,61,62,63,64,65], potentially with comorbidities of atopic and non-atopic systemic diseases [66].

A systematic review and meta-analysis of studies assessing AD prevalence found similar prevalence of atopy in childhood and adolescence/early adulthood, suggesting that skin barrier and immune dysfunction may persist into adulthood [62]. A multi-stage genome-wide association study on infantile eczema followed by childhood asthma revealed two novel loci specific for the atopic march phenotype (rs9357733 located in *EFHC* on chromosome 6p12.3 (OR = 1.27 [1.13–1.46]) and rs993266 between *TMTC2* and *SLCA16* on chromosome 12q21.3 (OR = 1.58 [1.27–1.96]). Other loci were also associated with eczema alone or asthma alone, which illustrate the heterogeneity of disease mechanisms underlying the atopic march [67].

Loss-of-function mutations in the filaggrin gene do not increase the risk of food or aeroallergen sensitivity independently of AD status [68,69], suggesting there must be other important genetic and/or environmental modifiers such as allergens, environmental pollutants, and oxidative stress that are required for the development of allergic skin inflammation, generalized T2 inflammation, and progression of the atopic march [8,70]. Allergens and irritants via the impaired skin barrier can lead to the release of alarmins (TSLP, IL-25, IL-33) which activate immature DCs and group 2 innate lymphoid cells (ILC2s) [70]. DCs then process the allergens, present them to naïve T cells, and induce Th2 differentiation. [70] Additionally, ILC2 cells contribute to an antigen-non-specific Th2 skewing [70,71,72]. Overall, the extensive activation of the Th2 pathway towards food and environmental allergens due to leaky epithelial barriers may secondarily lead to excessive B cell class switching to IgE, to endo- and exogenous antigens and pathogens [73]. In addition to T cell skin infiltration causing AD, concurrent redistribution of memory T cells from the skin into the circulation, gut, lung, and nose may thus initiate the atopic march from AD to FA, asthma, and eventually, AR [70,73].

**Figure 1:**
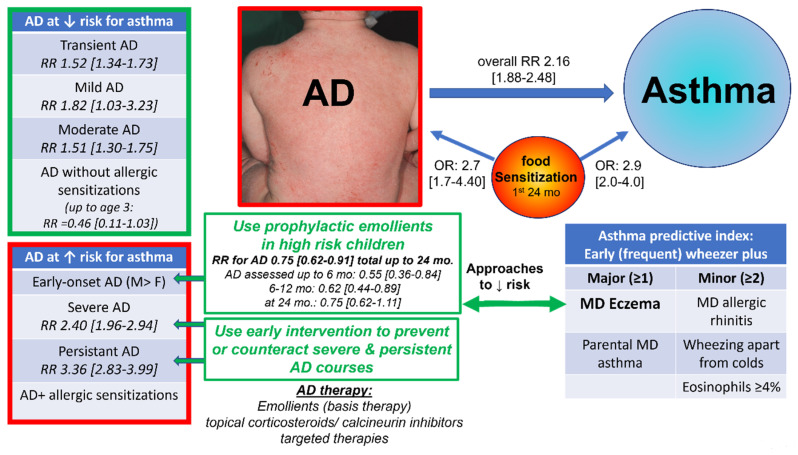
Atopic dermatitis with risk factors for the development of asthma, and implications for prophylactic and therapeutic intervention. Prophylactic emollients may contribute to the prevention or, at least, delay the early onset of AD within the first year of life. AD with early-onset, severe, and persistent disease course are at a higher risk for asthma. Early intervention and adequate treatment of AD might prevent the progress from mild and transient AD to severe and persistent forms, and in already existing severe AD, might contribute to downregulation of the severity and persistence of AD. This, in turn, might reduce the risk of the development of asthma = 95% Confidence interval, MD= physician diagnosis, mo=months, OR = odds ratio, RR = risk ratio. Effect sizes of risk factors [14,74], and the Asthma Predictive Index (API) estimating the probability of the development of asthma in a child with a history of wheezing [75,76,77], have been adapted from the literature. The Table with the API has been modified and adapted from: JOSÉ A. CASTRO-RODRÍGUEZ , CATHARINE J. HOLBERG, ANNE L. WRIGHT , and FERNANDO D. MARTINEZ/2000/ A Clinical Index to Define Risk of Asthma in Young Children with Recurrent Wheezing/Am J Respir Crit Care Med/Vol 162. pp 1403–1406, 2000. Adapted with permission of the American Thoracic Soci-ety. Copyright © 2022 American Thoracic Society. All rights reserved. The American Journal of Respiratory and Critical Care Medicine is an official journal of the American Thoracic Society. Readers are encouraged to read the entire article for the correct context at https://www.atsjournals.org/doi/full/10.1164/ajrccm.162.4.9912111. The authors, editors, and The American Thoracic Society are not responsible for errors or omissions in adaptations.

AD, overall, had a risk ratio (RR) for subsequent asthma in AD of 2.16 (95% CI, 1.88–2.48) in a recent meta-analysis of 39 publications with a total of 458,810 participants (Figure 1) [74]. Stratification revealed a higher risk of asthma in persistent and severe AD than in transient, mild or moderate forms (RR: transient AD = 1.52 [1.34–1.73], persistent AD = 3.36 [2.83–3.99], mild AD = 1.82 [1.03–3.23], moderate AD = 1.51 [1.30–1.75], severe AD = 2.40 [1.96–2.94]). Furthermore, early-onset AD had a slightly higher risk than late-onset AD, and boys were a higher risk than girls [74]. Therefore, AD with early-onset, severe, and persistent disease courses, especially, should be closely monitored for concomitant asthma in an interdisciplinary approach, considering the increased risk of these phenotypes. Early intervention and adequate treatment of AD might prevent the progress from mild and transient AD to severe and persistent forms, and in already existing severe AD, might contribute to downregulation of the severity and persistence of AD. This, in turn, might reduce the risk of the development of asthma.

AD is also one major criterion of the Asthma Predictive Index (API) estimating the probability of the development of asthma in a child with a history of wheezing (Figure 1) [75,76,77]. The stringent API predicts a higher likelihood of asthma if the following criteria are met: frequent episodes of wheezing up to age 3 years (early frequent wheezer) plus at least 1 major (physician-diagnosed AD/ eczema, parental history of physician-diagnosed asthma) or 2 minor criteria (physician-diagnosed allergic rhinitis, wheezing apart from colds, peripheral eosinophils ≥4%,) (Likelihood-ratio 7.3 for asthma at age 6–8). Frequent wheezing was defined as ≥3 on a 5-point scale, 1 “very rarely” to 5: “on most days” (parental report). The loose API is defined as any early wheezing up to age 3 years plus the same criteria and has a higher sensitivity, but lower specificity [75,76,77]. Several birth cohort studies support these criteria [75,76,77].

A longitudinal Canadian birth cohort study including 2311 children did not find a generally increased risk of asthma up to age 3 years in AD without allergic sensitization (RR = 0.46 [0.11–1.93]) [78]. Conversely, AD with allergic sensitization had a more than 7-fold (RR = 7.04 [4.13–11.99]) increased risk for asthma and 15-fold increased risk for FA (RR 15.11, 95% CI 4.19–35.36) with relative excess risk due to interactive effects on both asthma and FA [78]. A meta-analysis of 13 cohorts detected early food sensitization in the first 2 years as a risk factor for wheeze/ asthma (OR = 2.9 [2.0–4.0]), eczema (OR = 2.7 [1.7–4.4]), and AR (OR = 3.1 [1.9–4.9]) at age 4–8 years [79]. Analysis of the high-risk Melbourne Atopic Cohort Study (MACS) (*n* = 620) and the population-based LISA plus (*n* = 3094) confirmed the role of early food sensitization within the first 24 months on allergic airway diseases by age 10–12 years with stronger effects of cosensitization towards aeroallergens, both on subsequent asthma (MACS: aOR = 8.3 [3.7–18.8]; LISAplus: aOR = 14.4 [5.0–41.6]) and AR (MACS, aOR = 3.9 [1.9–8.1], LISAplus 7.6 [3.0–19.6]) [80]. However, follow-up data of birth cohort studies into adolescence and to adulthood is limited [79]. Two large longitudinal birth studies found an association of all AD courses with asthma with the strongest associations for early-onset persistent AD (asthma at age 7: OR = 14.27 [7.33–27.78] (PIAMA), OR = 5.50 [4.28–7.05] (ALSPAC); at age 11: OR = 15.35 [6.86–34.35] (PIAMA); at age 13: OR = 7.19 [5.48–9.42] (ALSPAC), (ALSPAC: *n* = 9894, PIAMA: *n* = 3652) [81].

Moreover, for FA, an increased risk could be shown for severe forms of AD in adults (mild AD: RR = 1.48 [0.89–2.07], moderate AD: RR = 2.40 [1.54–3.27], severe AD: RR = 8.49 [5.44–11.54] compared to the general population), yet with a significant heterogeneity across the studies [82].

There is growing data regarding the associations of moderate-to-severe forms of AD in adulthood, also with non-atopic comorbidities, potentially reflecting a high level of systemic immune activation (T cells, B cells, and circulating cytokines) [66,82,83,84]. The immunological changes also in non-lesional AD skin indicate inflammation beyond clinical visible inflamed lesions [34,85]. Epidemiologic studies have revealed a positive correlation between AD and systemic conditions, such as rheumatoid arthritis, inflammatory bowel disease, and neonatal adiposity [86]. Furthermore, adult AD has been associated with other dermatological comorbidities such as urticaria (OR = 9.92 [6.43–15.32] [82,87] and alopecia areata (AA) (OR = 25.31 [14.48–47.80] [82,88,89], with moderate and severe AD increasing the probability of AA compared to mild AD [90]. Data on the association of AD with cardiovascular diseases are heterogeneous with, overall, very small associations, mainly in severe AD, e.g., mild AD is not a statistically significant risk factor for myocardial infarction but severe AD slightly increases the risk (Hazard Ratio = 1.37 [1.12–1.68]) [82,84].

The association between AD and neuropsychiatric conditions has also been widely studied, with an increased risk of mental health disorders such as depression [OR = 1.99 [1.53–2.59] [82] and anxiety [OR = 1.40 [1.12–1.75] [82], both strongly influenced by sleep disorders [29,91]. Psychological stress often aggravates AD, while the symptoms (pruritus, lack of sleep) can also act as secondary stressors and lead to a deterioration in quality of life [91], up to suicidal ideation (OR = 1.71 [1.43–2.03]) [82]. Psychological stress is also associated with abnormal skin barrier function and a shift toward cytokine expression in Th2 cells [92,93].

Alternatively, or in addition, there might be an innate predisposition or susceptibility to have more than one atopic disorder due to dysregulation of the immune system, shared genetic loci or early environmental triggers and microbiome dysregulation [94,95,96,97]

## 4. Implications for Preventive and Therapeutic Interventions

Previously, management of AD focused on avoiding triggers, managing exacerbating factors, emollient therapy (so-called “basis therapy”) for improving skin hydration and barrier function, antimicrobial therapy, and topical or systemic anti-inflammatory therapy depending on the severity of each individual case. Effective management of AD requires a multi-pronged approach to restore the skin barrier function, to reduce the inflammation, and to correct dysbiosis [13,98]. A consensus-based European guideline recently reviewed avoidance strategies, basic emollient treatment and bathing, dietary intervention, topical anti-inflammatory therapy such as topical corticosteroids and topical calcineurin inhibitors (tacrolimus, pimecrolimus), phototherapy and antipruritic therapy [99], antimicrobial therapy, systemic treatment, allergen-specific immunotherapy, complementary medicine, psychosomatic counseling, and educational interventions [100]. Newer strategies are moving from symptom control to potentially modifying disease using biologics to target individual inflammatory pathways [3,101,102,103].

Underlying mechanisms of the systemic impact of the disease in AD may explain why certain patients respond better to certain treatments and highlight the importance of precision medicine [3,102]. The identification of alterations in gene, protein, and lipid expression patterns, as well as the microbiome, will enable more sophisticated endotyping of early AD to help predict children at risk for AD and its comorbidities, thus allowing early institution of more focused pathogenesis-driven preventive interventions [97,104,105]. An extensive review has recently been published by the International Eczema Council on the use of biomarkers in AD, and large-scale clinical trials using minimal techniques, such as tape-strips [106], are now warranted to facilitate AD research and improve patient management [33]. The stratification of neonatal/pediatric AD patients into distinct biomarker-based endotypes [107,108,109] will contribute to more age-specific and personalized treatment approaches with biologicals targeting immune pathways or new targets such as epithelial components and epigenetic modifications [13,104]. For example, in adults with resolved AD, the presence of long-term immune-modifying alterations in melanocytes, and upregulation of possible anti-inflammatory markers such as PLA2G7 compared to healthy control skin, may provide the basis for developing prognostic disease parameters and biomarkers for therapy responses in AD [110].

### 4.1. Preventive Strategies Based on Skin Barrier Dysfunction: Precision Prevention with Dermocosmetics

Skin care products are cosmetics by regulatory definition. They may be used as prophylactic emollients to prevent eczema or as therapeutic emollients such as “basic therapy” in cases of already existing eczema. Emollients represent an important pillar in the management of AD symptoms and help to achieve disease control as dry skin is a key feature of the disease. They may also be used to modulate the skin microbiome [111]. They are potentially useful to prevent or delay the emergence of AD by improving skin barrier integrity and blocking the inflammatory cascade of the atopic march [21,23]. Evidence of skin barrier dysfunction in non-lesional skin of children with AD suggests that skin care should be instigated early. Although most moisturizers showed some beneficial effects, a number of studies have evaluated the efficacy of emollients in preventing AD, with mixed results [112,113,114].

Three different pilot, randomized, controlled trials (RCT) in high-risk neonates showed that standard emollient (two studies) or ceramide-dominant emollient (one study) therapy from birth represents a feasible, safe, and effective approach for reducing the incidence of AD by approximately 50% [115,116,117]. A recent meta-analysis analyzed the effects of prophylactic emollients initiated within the first six weeks of life on the development of AD by age 24 months and on FA compared to no treatment [14]. Study heterogeneity limiting comparison of results from different studies was assessed with *I*^2^ values (0–40% low, 40–75% substantial, 75–100% considerable heterogeneity). Prophylactic emollients showed no protective effect against food sensitization to any food (egg, milk, peanut) (RR = 1.10 [0.83–1.46], *I*^2^ = 40%) (*n* = 1455 from 5 RCT). The efficacy of prophylactic emollients for AD prevention depended on the patient’s risk, age at outcome assessment, and treatment duration [14]. Emollients had a preventive effect against AD in high-risk children with a positive family history for atopy (RR = 0.75 [0.62–0.911], *I*^2^ = 10%) (*n* = 2059 from 8 RCT). The risk reduction was statistically significant without study heterogeneity at AD assessment up to 6 months (RR = 0.55 [0.36–0.84], *I*^2^ = 0%), and at age 6–12 months (RR = 0.62 [0.44–0.89], *I*^2^ = 0%), but not statistically significant at 24 months (RR = 0.92 [0.76–1.11]). The authors hypothesized that the prophylactic emollients may attenuate deleterious effects of increased TEWL in early life, but later genetic and environmental factors might contribute to delaying rather than completely preventing onset of AD [14]. Furthermore, continuous use of emollients up to the point of AD assessment showed a benefit (RR = 0.59 [0.43–0.81], *I*^2^ = 0%), but not if treatment was discontinued before AD assessment (RR = 1.11 [0.80–1.54], *I*^2^ = 68%). In the broad analysis of all patients without stratification, emollients still showed a protective effect up to the age of 6 months (RR = 0.55 [0.36–0.85], *I*^2^ = 0%). However, there was a substantial study which showed heterogeneity and no significant protective effect on the development of AD up to 24 months (RR = 0.84 [0.64–1.10], *I*^2^ = 60%) (*n* = 3505 from 10 RCT) compared to controls [14], highlighting the importance of stratification in both the analysis and clinical translation in precision medicine.

Another recent Cochrane meta-analysis [114] used a different individual participant data approach with a less strict definition of the AD outcome and included broader skin interventions (including bathing products) starting before the age of 12 months (rather than age <6 weeks [14]). Of 33 RCTs, 17 trials (*n* = 5823) had relevant outcomes, including 13 studies on emollients [14,114]. This Cochrane meta-analysis concluded that infant skin care interventions such as emollients during the first year of life in healthy infants are probably not effective for preventing AD and might even increase risk of skin infection [114]. A cluster RCT in 2697 women, evaluating early skin emollients (bath additives and facial cream), early complementary feeding (peanut, cow’s milk, wheat, egg), or both combined, did not support the use of these interventions to prevent AD by 12 months of age in infants [118]. In the BEEP RCT, in 1394 high-risk newborns, daily use of emollients during the first year of life showed no preventive effect on AD, as assessed at 24 months of age, and an increased risk of skin infections [119]. However, infections were a parent-reported outcome without objective ascertainment including all bacterial, fungal, and viral infections complicating pathological associations with emollient applications [14]. Future clinical trials with pathogen-specific physician-diagnosed infections are warranted to further clarify a potential association between emollients and skin infections [14]. The authors of the BEEP study commented that newer, improved, emollient formulations might potentially exert a protective effect, which could be enhanced if accompanied by additional measures such as soft water and avoidance of soap. Furthermore, the meta-analysis detected significant heterogeneity across the studies in type of emollient used, frequency (4×/week to 3×/day), duration, and body area of application (face only to full body) [14]. Studies reporting significant benefit of prophylactic emollients mostly reported at least daily application to the majority of the skin surface. However, potential additional benefits of these measures could not be quantified across the studies due to lack of specification in some studies [14]. Further potential confounders might be low adherence to the study protocol in the intervention group and use of emollients in the control group [14].

Traditional emollients are topical treatments with vehicle-type substances lacking active ingredients, whereas newer emollients, referred to as “emollients plus” in the European guideline, contain vehicle-type substances and putative active, non-medicated substances for topical treatment of AD [99]. Putative active ingredients of “emollients plus” include flavonoids and riboflavins from protein-free oat plantlet extracts, bacterial lysates from *Aquaphilus dolomiae* or *Vitreoscilla filiformis* species, or a synthetic derivative of menthol [120]. Other formulations include prescription emollient devices (medical device creams), which are designed to target specific defects in skin barrier function observed in AD [121]. A recent systematic review (including 29 guidelines) found that, while therapeutic recommendations and selection criteria for the type of emollient differed across the guidelines, the selection factor mentioned most often was patient preference, which is important to ensure good adherence, as emollients should be used liberally and frequently [122].

New emollient formulations for skin barrier repair and prevention of AD could have the following properties: (i) lipid ratios similar to the skin’s natural lipid composition; (ii) moisturizing agents (humectants, ceramides, emulsifiers); (iii) occlusive agents; (iv) pH similar to that of skin (pH 4–6) [123,124], and (v) probiotic fragments, as well as other ingredients, to reduce *Staphylococcus* biofilm formation and prevent microbiome dysbiosis [125]

Novel local therapies of dermocosmetics or emollients to promote microbiome diversity have recently been discussed [3]. Briefly, therapies include prebiotics and probiotics, emollient-mediated microbiome changes supplemented with *Vitreoscilla filiformis* or *Aquaphilus dolomiae*, or microbiome transplantation topical application of commensal organisms (e.g., *Staphylococcus hominis* or *Roseomonas mucosa*) [3].

In summary, the prophylactic and continuous application of emollients may prevent or only delay the onset of AD in high-risk populations [14], with heterogeneity of studies impeding overall generalizability. Future studies with detailed information of the participants’ phenotype, physician-assessed disease diagnosis and severity, epidemiological and biomarker data, while considering emollient composition, frequency, duration, and body area of application [14] are warranted to clarify this issue. Conflicting results on the effectiveness of emollients for prevention in newborns indicate that there is no “one-size fits all” in prevention since the mechanisms of barrier dysfunction are so diverse, and a more “precise” approach with further data is needed, both for prophylactic use and therapeutic use of emollients in clinically manifested AD.

### 4.2. Therapeutic Interventions: Precision Medicine

Besides the core T2 inflammatory cytokines (IL)-4, IL-13, and IL-31, a variety of other mediators have been reported to be instrumental depending on the age of the patient, the ethnic background, the course, and the duration in AD. Translational research has led to the development of various biological therapies and small molecules targeting these cytokines for systemic immunomodulation of AD [3,10,126,127,128,129]. Table 1 gives an overview of systemic targeted therapies approved or in clinical trials for atopic dermatitis (AD) and asthma, respectively.

Blocking the IL-4/IL-13 pathway shows efficacy for treating the inflammatory aspects of many, but not all, patients with moderate-to-severe AD, stressing the need for precision medicine with stratification by comorbidities and other phenotypic traits as well as the endotype [3,130].

IL-13 is not only crucial for T2 inflammation in AD, but also in allergic asthma contributing to goblet cell hyperplasia, smooth muscle contractility, and isotype switch of B cells towards IgE [131,132].

**Table 1:** Overview of systemic targeted therapies approved or in clinical trials for atopic dermatitis (AD) and/or asthma.

Target	Agent	AD *		Asthma		Other Indications	Remarks for AD and Asthma
		Approv-ed	RCT	Approv-ed	RCT		
**Adaptive immune response**							
**IL-4Rα**	**dupilumab**	**+ (≥6 y.)**	**a.**	**+ (≥6 y.)**	**a.**	**CRSwNP (add-on),** RCT for AR, pruritus, FA, CHP, keloid, HE, AA, AERD, nummular eczema, EE, EG, sinusitis, metastatic non-small cell lung cancer, prostate CA, sleep apnea, cold urticaria, scleredema, Netherton sd, BP, COVID-19, peanut allergy	Severe T2 asthma (eosinophils ≥ 150/µL, FeNO > 25 ppb). Add-on maintenance therapy
**IL-4Rα**	**CBP-201**	-	IIb	-	II	RCT for CRSwNP	
**IL-4Rα**	**AK 120**	-	Ib	-	II	-	
**IL-13**	**tralokinumab**	+ (≥18 y.)	a.	-	III	RCT for ulcerative colitis, AA, idiopathic pulmonary fibrosis	↑ response for subgroups with ↑ levels of periostin, DPP-4, IL-13, inconsistent results for asthma overall
**IL-13**	**lebrikizumab**	-	III	-	II	RCT for COPD, idiopathic pulmonary fibrosis	↑ response for subgroups with ↑ levels of periostin
**IL-13Rα1**	**eblasakimab**	-	IIb	-	-	-	
**IL-5**	**mepolizumab**	-	-	**+ (≥6 y.)**	**a.**	**CRSwNP(add-on),** HES, EPGA RCT for eosinophilia, COPD, eosinophilic fasciitis, esophagitis, angioedema, CSU	Severe eosinophilic asthma (add-on)
**IL-5Rα**	**benralizumab**	-	II	**+ (≥18 y.)**	**a.**	RCT for EG, non-cystic fibrosis bonchiectasas, CRwNP, HES, nasal polyps, COPD, skin side effects caused by cancer therapy, eosinophilic chronic rhinosinusitis, cystic fibrosis	Severe eosinophilic asthma
**IL-5Rα**	**reslizumab**	-	-	**+ (≥18 y.)**	**a.**	RCT for sinusitis, EE, HES	Severe eosinophilic asthma (add-on)
**IgE**	**omalizumab**	-	II	**+ (≥6 y.)**	**a.**	**CRSwNP (add-on), urticaria (CSU)**, RCT for FA, immunotherapy, BP, SLE, AR, Sjogren´s sd, mastocytosis, EE, cholinergic U., solar U., AE anaphylaxis, COPD, CF, HES, Job´s Sd, interstitial cystitis, ASS hypersensitivitiy	Allergic asthma AD: program discontinued
**IgE**	**FB825/anti-CεmX**	-	IIa	-	II	-	AD: program discontinued
**Histamine**							
**H4R**	**adriforant**	-	IIb	-	-	-	AD: program discontinued
**H4R**	**LEO152020/JW1601**	-	IIb	-	-	RCT for cholinergic urticaria	
**Other**							
**IL-22**	**fezakinumab**	-	IIa	-	-	RCT for RA, psoriasis	
**IL-22R1**	**LEO 138559**	-	Ib	-	-	-	
**IL-17A**	**secukinumab**	-	IIa	-	-	**Plaque psoriasis, psoriatic arthritis**, axial spondyloarthritis, RCT for HS, psoriasis, discoid LE, necrobiosis lipoidica diabeticorum, pyoderma gangrenosum, autoimmunity	AD: program discontinued
**IL-23**	**risankizumab**	-	IIa	-	-	**Plaque psoriasis, psoriatic arthritis**, RCT for COVID-19, HS, AS, palmoplantar pustulosis, Crohn´s disease, ulcerative colitis, dermatitis	
**rhIL-2 to T_reg_ cells**	**Ly3471851**	-	Ib	-	-	RCT for psoriasis, SLE, ulcerative colitis,	
**OX 40**	**GBR 830/ISB 830**	-	IIb	-	-	-	
**OX 40**	**KHK 4083**	-	IIb	-	-	RCT for ulcerative colitis, digestic system diseases	
**OX 40**	**KY1005**	-	IIa	-	-	RCT for immune system diseases	
**CCR4**	**RPT193**	-	IIa	-	-	-	
**S1PR1,4,5**	**etrasimod**	-	IIb	-	-	RCT for eosinophilic eophagitis, ulcerative colitis (III), Crohns´s disease, AA, PG	
**S1PR1**	**SCD-044**	-	IIb	-	-	RCT for plaque psoriasis	
**S1PR1**	**BMS-986166**	-	IIb	-	-	RCT for ulcerative colitis	
**S1PR1**	**LC51-0255**	-	I	-	-	RCT for ulcerative colitis	
**S1PR1**	**KT-474**	-	I	-	-	RCT for HS	
**Innate immune response**							
**TSLP**	**tezepelumab**	-	IIa	**+ (≥12 y.)**	**a.**	RCT for COPD (IIa), CSU (H2H with omalizumab), CRSwNP	Severe asthma (add-on), RCT for pediatric asthma ≥5–11 y (I), AD: discontinued, ↓ efficacy
**IL-33**	**etokimab**	-	IIa	-	-	RCT for CRSwNP	AD: IIa: primary end point not reached
**IL-33**	**Itepekimab #**	-	IIa	-	II	RCT for COPD (IIa)	IIa: improved asthma control, QoL, reduction of eosinophils
**Il-33**	**astegolimab**	-	IIa	-	-	RCT for COPD, COVID-pneumonia	
**IL-33**	**tozorakimab (MEDI3506)**	-	IIa	-	II	RCT for COPD, chronic bronchitis, diabetic kidney disease	
**IL-1α**	**bermekimab**	-	IIa	-	-	RCT for hidradenitis suppurativa (HS), systemic scleroderma, metastatic colorectal cancer, advanced cancers, type 2 diabetes	
**IL-36 R**	**spesolimab**	-	IIa	-	-	RCT for Crohn’s disease, HS, generalized pustular psoriasis, palmoplantar pustulosis, ulcerative colitis	AD: program discontinued
**Microbiome**							
**micro-biome**	**OM-85**	-	II	-	IV	**Prevention of recurrent and lower respiratory tract infections (bronchitis, sinusitis)**, RCT for COPD, bronchiectasias, sleep, pain, stress, adenoid hypertrophy/ hyperplasia, COVID-19, mucositis, stomatitis, uveitis, head and neck squamous cell cancer, solid tumors, hematologic malignancies, overweight, essential fatty acid defiency, hypercholesterolemia, hypertriglyceridemia, hypertension, Parkinson’s disease, RA, HIV, SLE, psychiatric disorders, metabolic syndrome	
**micro** **biome**	**EDP1815**	-	II	-	-	RCT for COVID-19, psoriasis,	
**micro-biome**	**STMC-103**	-	Ib	-	-	RCT for type 1 hypersensitivity, atopic IgE mediated allergic disorder	
**Pruritus**							
**IL-31R**	**nemolizumab**	-	III	-	-	RCT for PN, systemic sclerosis, chronic kidney disease-associated severe pruritus	Prurigo-form AD best responses, RCT also for pediatric AD (age 2-6 (II), 7-11 (II), 12-17 y (II))
**OSMRß**	**vixarelimab**	-	IIb	-	-	RCT for pruritus, PN, chronic idiopathic urticaria, lichen planus, lichen simplex chronicus, plaque psoriasis	
**NK1R**	**serlopitant**	-	II	-	-	RCT for pruritus, PN, psoriasis, refractory chronic cough, burns, epidermolysis bullosa	AD: program discontinued
**NK1R**	**tradipitant**	-	II	-	-	RCT for COVID-19, pastorparesis, motion sickness, pruritus	
**P2X3**	**BLU-5937**	-	II	-	-	RCT for pruritus, chronic (refractory) cough	
**Janus kinases**							
**JAK1/JAK2**	**baricitinib**	**+ (≥18 y.)**	**a.**	-	-	**RA,** RCT for AA, COVID-19, pneumonia, SARS, ACD, vitiligo, lichen planus, pyoderma gangrenosum, wound heal, dermatomyositis, systemic sclerosis, SLE, Sjogren´s syndrome, psoriasis, other skin diseases, polymylagia rheumatic, mypathies, uveitis, chronic graft vs. Host disease, type 1 diabetes, diabetic kidney disease, liver diseases, hepatic insufficiency, arteritis (giant cell), ALS, Alzheimer’s disease, systemic sclerosis, NNS/CANDLE, SAVI, AGS, ankylosing spondylitis, psoriasis arthritis	
**JAK1**	**upadacitinib**	**+ (≥12 y.)**	**a.**	-	-	**RA, psoriatic arthritis, ankylosing spondylitis**, RCT for SLE, juvenile idipathic arthritis, ulcerative colitis, Crohn´s disease, arteriits (Takayasu, giant cell), non-segmental vitiligo,	
**JAK1**	**abrocitinib**	**+ (≥18 y.)**	**a.**	-	-	-, RCT for FA, PN, pruritus, psoriasis, renal impairment	
**JAK1**	**SHR0302**	-	II	-	-	-, RCT for RA, AS, PsA, AA, GVHD, vitiligo, ulcerative colitis, primary membranous nephopathy	

Blue: approved (a., +) for AD and/or asthma, Bold: Other approved indications are printed in bold. RCT = randomized controlled trial, with the respective phase of the drug development program. AA = Alopecia Areata, ACD = allergic contact dermatitis, * AD with indication for systemic therapy according to the current guidelines, add-on: add-on maintenance treatment, AE = Angioedema, AERD = Aspirin Exacerbated Respiratory Disease, AGS = Aicardi Goutières Syndrome, ALS = amyotrophic lateral sclerosis, AR = allergic rhinitis, AS = ankylosing spondylitis, BP = bullous Pemphigoid, CANDLE = chronic atypical neutrophilic dermatosis with lipodystrophy and elevated temperature syndrome, CHP = chronic hepatic pruritus, CRSwNP = Chronic rhinosinusitis with nasal polyps, COPD = chronic obstructive pulmonary disease, CSU = chronic spontaneous urticaria, EE = eosinophilic Esophagitis, EG = eosinophilic gastritis, EPGA = eosinophilic granulomatosis with polyangiitis, FA = food allergy, GVHD = Graft versus host disease, HE = Hand eczema, HES = hypereosinophilic syndrome, H2H = head-to-head study, H4R = histamine receptor 4, HS = Hidradenitis Suppurativa, IL = Interleukin, JAKi = janus kinase inhibitor, mab = monoclonal antibody, NK1R = neurokinin 1 receptor, NNS = Nakajo–Nishimura syndrome, OSMRß = oncostatin M receptor-ß, OX40 L = OX40 ligand, PN = prurigo nodularis, RA = rheumatoid arthritis, SARS = severe acute respiratory syndrome, SAVI = STING-associated vasculopathy with onset in infancy, SLE = Systemic Lupus Erythematosus, y = years, #: Itepekimab = REGN3500/SAR440340). References: https://clinicaltrials.gov, assessed on 13 May 2022, [3,133], assessed on 13 May 2022.

An appropriate therapy for AD with atopic comorbidities is dupilumab, a monoclonal antibody (mab) targeting the IL-4Rα which inhibits both IL-4 and IL-13 signaling. It is approved for moderate-to-severe AD in adults and children ≥6 years; as an add-on therapy for patients ≥6 years with severe asthma with type-2 inflammation, characterized by increased serum eosinophils (≥150/µL), elevated nitric oxide (FeNO > 25 ppb), insufficiently controlled by inhaled corticosteroids plus another maintenance therapy, and as add-on therapy for chronic rhinosinusitis with nasal polyps (CRSwNP), insufficiently controlled with systemic corticosteroids and/or surgery. In addition to other immunological and clinical effects, normalization of the skin microbiome could also be shown under therapy with dupilumab with a decrease of abundance of *S. aureus* and an increase in microbial diversity, and the abundance of *Cutibacterium* and *Corynebacterium species* [134,135].

Other potential candidates for both AD and asthma are the anti-IL-13 mabs, tralokinumab and lebrikizumab. Tralokinumab has recently been approved for treatment of moderate-to-severe AD with indication for systemic therapy, and lebrikizumab is currently in phase III clinical trials for AD [3]. The effects on asthma are controversial with insufficient efficacy in the overall study populations. In the phase III trials, tralokinumab reduced AAER in participants with severe asthma with baseline FENO ≥37 ppb in STRATOS 1, but not in STRATOS 2 [136]. However, both lebrikizumab and tralokinumab showed better response in subgroups of patients with high levels of periostin [131,137]. Periostin is induced by IL-13, links T2 inflammation with airway remodeling in the lung and keratinocyte activation in the skin, inducing production of proinflammatory cytokines which correlates with eosinophil levels, and is supposed to contribute to tissue remodeling and chronicity in both AD and asthma [138].

Other therapeutic targets for both AD and asthma are the alarmins TSLP and IL-33 [3], released from barrier tissues which activate the innate immune response [139,140]. The anti-TSLP mab, tezepelumab, has been approved in severe and uncontrolled asthma with significant reductions of exacerbations and improvements in lung function, symptom control and health-related quality of life, and is currently in phase III trials for asthma [140]. Conversely, results of a phase IIa study in AD were less convincing which might partly be due to the study design with use of topical corticosteroids in all patients [3]. Anti-IL-33 mabs in clinical trials for AD and/or asthma are etokimab (ANB020), itepekimab (REGN 3500), astegolimab (MSTT1041A/ AMG282), and tozorakimab [3]. In asthma, itepekimab improved lung function, asthma control and quality of life, and led to a greater reduction of eosinophils compared to placebo [133,141]. Results of RCTs with itepekimab and astegolimab for AD have not been published yet. While a proof-of concept study with etokimab showed promising results (EASI 50 in 83% of patients, EASI 75 in 33%, *n* = 12) [142], the primary end point was not reached in a phase IIa study with 300 AD patients [3]. Other biologics approved for severe T2 asthma targeting anti-IL-5 mab (mepolizumab, benralizumab, reslizumab for eosinophilic asthma) or the anti-IgE ab omalizumab have not shown sufficient efficacy in AD for approval [3]. Further strategies for both AD and asthma are aimed at modulating the microbiome. In addition to topical preparations, several systemic therapies (OM-85, EDP1815, STMC-103) are in ongoing clinical trials. Oral OM-85, an extract of bacterial lysates from 21 respiratory pathogenic strains is approved for the prevention and treatment of bronchitis and sinusitis, and is protective against airway infection of bacterial and viral origin [143]. Airway administration of OM-85 blocked experimental asthma in mouse models by targeting dendritic cells and the epithelium/ IL-33/ILC2 axis [144]. Children (aged 6 months to 7 years) with AD receiving adjuvant oral treatment with OM-85 showed fewer and delayed flares compared to placebo (*n* = 170) [145].

Other biologics approved for severe T2 asthma targeting anti-IL-5 mab (mepolizumab, benralizumab, reslizumab for eosinophilic asthma) or the anti-IgE ab omalizumab have not shown sufficient efficacy in AD for approval [3].

Therapeutic approaches that restore homeostasis or interrupt the flow of immunologic or neurosensory information may be of benefit for particular itches [46,47]. Other novel systemic therapies approved or in clinical trials for AD, but not asthma, target IL-31, OSMRß, NK1R, P2X3, H4R, IL-22, IL-17A, IL-23, OX40, CCR4, S1P, and Janus kinases [3]. JAK pathways are involved in signaling of several AD-related cytokines such as IL-4, -13, and -31 that mediate downstream inflammation and barrier alterations. Small molecule JAK inhibitors target different combinations of kinases with overlapping but distinct mechanisms of action, as reviewed recently [27,146]. JAKi have a rapid mode of action and showed high efficacy on pruritus and eczema in AD with recent approval of the JAKi baricitinib, upadacitinib, and abrocitinib. Baricitinib and upadacitinib are also approved for rheumatoid arthritis (RA), and there are several clinical trials ongoing for other inflammatory and autoimmune diseases such as AA, systemic lupus erythematosus, and others (Table 1) [3]. The broadening of the therapeutic landscape increasingly enables physicians to choose tailored therapies under consideration of comorbidities and side effects. AD patients with rheumatoid diseases or AA as frequent comorbidities of AD are supposed to profit most from JAKi, and patients with concomitant atopic diseases such as asthma or CRSwNP from dupilumab, especially in cases of eosinophilia.

A better understanding of the distinct mechanisms underlying the “immunologic march” in the natural course of AD and their contribution to the atopic march is crucial to design new therapeutic approaches aimed to not only treat the repetitive flares but, most importantly, to have properties of disease modification, and to hamper the progress into the atopic march in putative subgroups of patients at high risk of developing these comorbidities.

## 5. Conclusions and Future Perspectives

Combining precision diagnosis and early targeted therapies should help to address the heterogeneity of the disease. In high-risk children, continuous use of prophylactic emollients starting within a critical window of the first six weeks of life has been shown to reduce the probability of early-onset AD. Furthermore, therapeutic precision dermocosmetics, and local and systemic therapies have a meaningful role to play in managing AD for relief of symptoms, and potentially, a disease-modifying role to prevent progression to the atopic march. Severe and persistent phenotypes of AD have been shown to have a higher risk for asthma than mild, moderate, and transient forms. Early intervention and adequate treatment of AD might prevent the progress from mild and transient AD to severe and persistent forms and, in already existing severe AD, contribute to downregulation of the severity and persistence of eczema. Thus, early therapy of AD to restore the skin barrier and microbiome, and/or targeting the T2 inflammation, such as dupilumab, depending on the (endo)phenotype, is not only crucial for AD disease control, but might also contribute to obviating comorbidities. The stratification of neonates, children, and adult AD patients into biomarker-based endotypes to distinguish the different biochemical forms of skin barrier dysfunction, immune dysfunction, or microbial dysbiosis might allow them to be corrected using a tailored approach. An interdisciplinary precision medicine approach translating new immunological insights and lessons learned from birth cohort studies (with and without intervention), as well as from the varying responses to targeted therapies in clinical trials and registries to clinical practice in pediatrics, dermatology, pulmonology, otorhinolaryngology, and allergology is warranted. A better awareness of shared pathomechanisms and treatment options for AD and comorbidities may have practical consequences in terms of (i) choice of (add-on) therapy on the physician’s side and (ii) better compliance on the patient’s side after communication of the rationale for a particular targeted therapy. This will offer opportunities to introduce better long-term control of AD with the potential to reduce the systemic impact of the cutaneous inflammation, and possibly, even prevent or modify the course of not only AD, but also of other comorbidities depending on the patient’s age, disease stage, and type of comorbidities.

## Data Availability

Not applicable.
